# The study of psychological traits among Chinese college students during the COVID-19 campus lockdown

**DOI:** 10.3389/fpsyg.2022.1051770

**Published:** 2022-11-21

**Authors:** Haibo Xu, Zhen Wang, Lixin Peng, Yanyan Mi, Ping Zeng, Xin Liu

**Affiliations:** ^1^Center for Mental Health Education and Research, Xuzhou Medical University, Xuzhou, China; ^2^School of Management, Xuzhou Medical University, Xuzhou, China; ^3^Department of Epidemiology and Biostatistics, School of Public Health, Xuzhou Medical University, Xuzhou, China

**Keywords:** interpersonal sensitivity, anxiety, depression, the COVID-19 pandemic, campus lockdown

## Abstract

To investigate the prevalence of interpersonal sensitivity, anxiety, depression symptoms and associated risk factors among a large-scale sample of college students in China during the COVID-19 campus lockdown. The survey was conducted among undergraduate students at a university in eastern part of China in April 2022. The Chi-square test was used to compare the different variable groups and multivariable analysis was performed for the risk factors associated with interpersonal sensitivity, anxiety, and depression symptoms. A total of 12,922 college students were included, with an average age of (20.96 ± 1.66) years. The prevalence of interpersonal sensitivity, anxiety and depression symptoms in this study was 58.1, 22.7, and 46.8%, respectively. Male (OR = 1.16, *p* < 0.001), 22–23 years (OR = 1.40, *p* < 0.001), freshman (OR = 1.35, *p* = 0.002), and non-only child (OR = 1.15, *p* < 0.001) were positively associated with interpersonal sensitivity. Male (OR = 1.20, *p* < 0.001), sophomores (OR = 1.27, *p* = 0.020) and seniors (OR = 1.20, *p* = 0.027) were positively associated with anxiety symptoms. Compared with female students, male students (OR = 0.89, *p* < 0.001) were less likely to have depression symptoms. 22–23 years (OR = 1.37, *p* < 0.001), sophomores (OR = 1.26, *p* = 0.009) and non-only child (OR = 1.11, *p* = 0.009) were positively associated with depression symptoms. In addition, college students aged 18–21 years, learning status, skipping breakfast, roommate relationship and sleep quality were associated with interpersonal sensitivity, anxiety and depression symptoms (all *p* < 0.05). The findings of this study suggest a high prevalence of interpersonal sensitivity, anxiety and depression symptoms among Chinese college students during the COVID-19 campus lockdown. Younger ages, low grades, poor dormitory relationship, negative learning status, skipping breakfast and poor sleep quality were the risk factors for college students’ mental health, which should be concerned by the relevant departments of school during the campus lockdown.

## Introduction

The COVID-19 pandemic, a global health issue with significant implications is a stressor, leading to different degrees of biological, psychological, social reactions, among adolescents and young people (Branquinho et al., 2020; Manivannan et al., 2021). To prevent the spread of the pandemic, many countries around the world have taken various measures, including lockdown of schools or staying at home orders and strict social distancing (Ren, 2020, Aknin et al., 2022). Lockdown, as a state-spatial means for infectious disease mitigation, can effectively reduce the impact on the overall scale of the outbreak of the COVID-19 (Ren, 2020, Khataee et al., 2021).

Lockdown is like a double-edged sword, bring about a series of negative effects while curbing the rapid development of the epidemic (Onyeaka et al., 2021, Butterworth et al., 2022). Long period of quarantine was associated with the negative psychological effects (Brooks et al., 2020). The adverse psychological and behavioral effects usually focus on psychological disorders, such as posttraumatic stress disorder (PTSD), anxiety and depression (Morganstein and Ursano, 2020). Studies have shown that lockdown can significantly increase the risk of individual’s mental illness, such as gaming disorder (Shrestha et al., 2020), interpersonal violence (Ghimire et al., 2020; Jung et al., 2020), stress (Simegn et al., 2021), insomnia (Amicucci et al., 2021), PTSD (Cao et al., 2022), and suicide (McLafferty et al., 2021). In addition to the impact of lockdown management and preventive measures, COVID-19 also has the characteristics of paroxysm, urgency, harmfulness, infectivity and uncertainty, and it tends to induce negative emotions such as public panic, fatigue, anxiety, fear, anger and despair (Hossain et al., 2020).

A study systematically assessed the psychological impact of the pandemic and found that lower age (≤40 years) is one of the factors related to the pain of the general population (Xiong et al., 2020). During the first national lockdown in Britain, age was associated with lower levels of COVID-19 anxiety (Elliott and Pfeifer, 2022). During the COVID-19 lockdown in Nigeria, being younger were associated with more depressive symptoms (Chinawa et al., 2022). In Saudi Arabia, a study during the COVID-19 lockdown showed that 18–19 years old had the highest anxiety symptoms (28.7%; Albagmi et al., 2021). In China, a longitudinal study showed that prolonged lockdown had some adverse impacts on mental health, especially among the respondents aged 12–21 years, who showed a higher psychological impact of pandemic (Wang et al., 2020). During the COVID-19 lockdown, college students had a higher risk of mental health disorders than non-students (Arsandaux et al., 2021). The long-term lockdown management has caused obvious psychological burden to college students. A report from the midwestern USA shows that mental health deteriorated during the school lockdown (Olson et al., 2021). The sudden reduction of social activities can easily lead to social anxiety among young people (Li, 2020). Studies have confirmed that young people pay more attention to social relations and participate in more social activities than people of other ages (Savcı and Aysan, 2019, Labrague et al., 2021, Labrague and Ballad, 2021). During the pandemic, they are seriously affected by school lockdown, which may lead to loss of contact with peers and friends, increase interpersonal sensitivity and other mental health problems (Majumdar et al., 2020, Labrague and Ballad, 2021). A systematic review, reported that the commonest psychological diseases for adolescents with the COVID-19 lockdown are anxiety and depression (Panchal et al., 2021). Social isolation has a negative impact on mental health, physical exercise and eating habits (Amatori et al., 2020). In a study of retired athletes, it was found that exercising in the morning or evening improved mental performance and reduced depression (Irandoust et al., 2019). Appropriate and daily physical activity may improve the mental health of college students during COVID-19 (Zhang et al., 2020). In addition, due to the lockdown, economic losses, the adverse impact of daily life, the cancellation of social activities, the decline of learning efficiency of distance online courses and the delay of exams, a large number of students may also experience more emotional distress (Cao et al., 2020).

The uncertainty associated with COVID-19 is seen as adding a destabilizing factor leading to the anxiety and challenges of existing radiotherapy students (Courtier et al., 2021). During the lockdown, a longitudinal study from France showed that people with anxiety increased their anxiety and depression symptoms by 6.73 times after the community lockdown (Andersen et al., 2021). Compared with students without a history of depression, students with depression have a greater increase in anxiety and stress during the lockdown (Husky et al., 2021). A study from the vast majority of college students in the United States reported the intensification of depression and generalized anxiety symptoms after COVID-19 lockdown (López-Castro et al., 2021). A longitudinal study showed that the suicide ideation of Chinese college students increased during the lockdown of COVID-19 (Huang et al., 2022). Adolescents with anxiety and depression have social, peer relationship and academic barriers, and the risk of dropping out of school is higher than that of their peers (Harb et al., 2002, de Lijster et al., 2018, Savcı and Aysan, 2019). Adolescence is an important period of psychosocial transformation, and young people are facing great psychological changes. The COVID-19 disease significantly increased the psychological disorders of college students, while lockdown and quarantine had a significant adverse impact on college students’ mental health and emotion in different countries (Kaparounaki et al., 2020, Commodari et al., 2021). Chinese college students have their own unique characteristics and face different pressures from other peers, such as learning knowledge and skills; cultivating intimacy, avoiding loneliness, and experiencing romantic relationship, and adapting to college life (Wen et al., 2022).

The outbreak of COVID-19 generates a strong sense of distrust, nervousness, fear and overreaction to people around them, which could aggravate the level of interpersonal sensitivity and isolation of individuals (Mei et al., 2022). The impairment of interpersonal relationship is closely related to anxiety, depression and loneliness (Mohammadian et al., 2018; Dziedzic et al., 2021). According to a study analysis, the COVID-19 lockdown affected the interpersonal relationship between music teachers and students, as well as sports coaches and athletes, reducing their contact frequency and leading to a series of negative emotions (Antonini Philippe et al., 2020). However, there is a lack of research on the related factors of interpersonal relationships damaged during the lockdown of college students.

Several predictive factors were identified from previous studies. Females were reported to be generally more likely to have psychological problems when compared to their male counterparts (Lei et al., 2020, Mazza et al., 2020, Woday Tadesse et al., 2021). Participants from the group (low grades) presented with more psychological symptoms (Nakhostin-Ansari et al., 2020, Tesfaye Kelemu et al., 2020), while a study also identified higher grade as an associated factor with greater anxiety and depressive symptoms (Chen et al., 2021). Additionally, a study from China showed that sophomores reported higher levels of anxiety symptoms (Sun et al., 2020). Academic activities and learning status were positively associated with symptoms of mental health problems during the pandemic (Hou et al., 2020, Arsandaux et al., 2021, Jiang, 2021, Peng et al., 2022). It is known that the absence of interpersonal communication would lead to and deteriorate mental disorders during quarantine (Xiao, 2020). College students with depression have difficulty in contacting their roommates and are more sensitive to interpersonal relationships (Hokanson et al., 1989). A study found that non-only child participants developed more depression and PTSD symptoms than only child (Cao et al., 2022). A survey of nursing students in Zhengzhou, China, revealed that whether they were only children or not was not related to the anxiety caused during the epidemic of COVID-19 (Sun et al., 2020). In addition, the increase of gastrointestinal symptoms during the lockdown period in a population of medical students, may be correlated to both dietary habits and anxiety (Abenavoli et al., 2021). Several studies also identified poor sleep quality levels as an associated factor with greater psychological problems during the lockdown (Huang and Zhao, 2020, Castellini et al., 2021, Bhat et al., 2022). Also, young respondents reported more serious sleep obstacles (Amicucci et al., 2021) and a study found that compared to before lockdown, sleep duration increased by 0.8 h in average (Nirala et al., 2022).

Therefore, this study aims to investigate the interpersonal sensitivity, anxiety and depression symptoms of college students during the COVID-19 epidemic campus lockdown, and explore the potential risk factors related to these symptoms, so as to provide important evidence for targeted intervention and promotion of college students’ mental health.

## Materials and methods

### Participants and procedure

The sample data in the present study was obtained from the Student Mental Health Center at a university in eastern China after 2 weeks of static management (April 10 to April 19, 2022). The static management requirements were as follow: students could carry out activities on campus according to their actual needs (such as dining, buying daily necessities). In the absence of special circumstances, students did not enter or leave the campus gate for mobility. A total of 12,922 college students were included in this study. College counselors was sent the link to the online questionnaire to WeChat groups for undergraduates to complete the questionnaire.

### Measures

The survey consisted of two sections. First, the social demographic and other relevant features of the undergraduate students were recorded *via* the questionnaire, including gender, age, grade, only child, learning status, dormitory relationship, eating breakfast, and sleep quality. Sociodemographic data were collected by self-designed questionnaires. Second, the survey involved three scales as the assessment tools (see below for details):

#### Interpersonal sensitivity

The interpersonal sensitivity subscale of symptom checklist 90 (SCL-90) was employed (Derogatis et al., 1976), among Chinese college students and with good reliability (Ding et al., 2021). The subscale consists of 9 items and each one rated from 0 (“no”) to 4 (“very severe”). A higher sum score of all items indicated more severe level of interpersonal sensitivity. In the present study, the Cronbach’s α of the scale was 0.958.

#### Anxiety

Anxiety symptoms was measured by the generalized anxiety disorder 7-item scale (GAD-7; Spitzer et al., 2006). To assess the anxiety symptoms of participants over the past 2 weeks. The scale was developed by Spitzer and included 7 items where each item rated from 0 (“never”) to 3 (“almost every day”). The sum score of 5, 10 and 15 represent thresholds for mild, moderate and severe anxiety symptoms, respectively. The GAD-7 has been used among Chinese students with good reliability and validity (Zhou et al., 2020c). In our study, the Cronbach’s α of the scale was 0.936.

#### Depression

The patient health questionnaire 9-item depression scale (PHQ-9) was used to measure the severity of depressive symptoms (Kroenke, 2021). PHQ-9 has been used among Chinese students and shown good reliability and validity (Zhou et al., 2020c). The scale consists of 9 items with each item rating from 0 (“never”) to 3(“almost every day”), and the total score of 5, 10, 15, and 20 represent thresholds for mild, moderate, moderately severe, and severe depressive symptoms, respectively. The Cronbach’s α of the scale was 0.911 in the present study.

### Statistical analysis

Descriptive statistics were used to present socio-demographic data, and the prevalence of symptoms of interpersonal sensitivity, anxiety, and depression was calculated. Depression and anxiety were classified as present (mild to severe) and absent (normal), while interpersonal sensitivity was classified as present and absent. The Chi-square test was used to compare the difference of interpersonal sensitivity, anxiety, depression symptom prevalence between exposed and non-exposed groups. To determine the factors associated with interpersonal sensitivity, anxiety, and higher levels of depression, bivariate logistic regression analyses were performed, and Odds Ratios (OR) and 95% confidence intervals (CI) were presented. *P* < 0.05 was considered statistically significant (two sided). All data shall be subject to SPSS 26.0 software package for statistical analysis.

### Ethical considerations

This study, a non-intervention study based on the sample database, was approved by the Ethics Committee of Xuzhou Medical University. All research methods were performed in accordance with relevant guidelines of Declaration of Helsinki. All samples’ information was authorized by the database owner for use in this study, and information’s desensitization was conducted before the study to protect the participants’ privacy. All participants in the questionnaire fulfilled the requirement of informed consent.

## Results

### Prevalence of interpersonal sensitivity, anxiety and depressive symptoms

The prevalence of interpersonal sensitivity, anxiety and depression symptoms were shown in Table 1. In terms of interpersonal sensitivity, 58.1% of the participants reported symptoms during the period of school lockdown. The overall prevalence of anxiety and depression symptom was 22.7 and 46.8% in college students during COVID-19 epidemic, respectively. Among them, 18.4% of college students had mild anxiety and 33.7% had mild depression.

**Table 1:** Overall scores and prevalence of interpersonal sensitivity, anxiety symptoms and depression symptoms [*n* (%)].

Variable	*n* (%)	Prevalence
**Interpersonal sensitivity (1–5 scores)**		7,502 (58.1)^a^
No	5,420 (41.9)	
Yes	7,502 (58.1)	
**Anxiety symptoms (1–21 scores)**		2,927 (22.7)^b^ 548 (4.2)^c^
Minimal	9,995 (77.3)	
Mild	2,379 (18.4)	
Moderate	314 (2.4)	
Severe	234 (1.8)	
**Depression symptoms (1–27 scores)**		6,051 (46.8)^b^ 1,697 (13.1)^c^
None	6,871 (53.2)	
Mild	4,354 (33.7)	
Moderate	982 (7.6)	
Moderately severe	421 (3.2)	
Severe	294 (2.3)	

^a^≥2 scores; ^b^≥5 scores; ^c^≥10 scores.

### Demographic characteristics and the detection rates of interpersonal sensitivity, anxiety and depressive symptoms among college students with different general data

Demographic characteristics based on presence/absence of interpersonal sensitivity, anxiety and depression symptoms were summarized in Table 2. A total of 12,922 responses were included. In terms of gender, 5,520 (42.7%) females and 7,402 (57.3%) males responded to the survey. The mean age of participants was 20.96 years (SD = 1.66). 72.8% of the participants were aged 20–23. The proportion of samples in each grade is relatively balanced. The majority of participants (56.3%) were in general learning status. Nearly half of the participants (50.4%) had good dormitory roommates. Approximately half of the sample (52.0%) were the only child. The percentage of participants who often ate breakfast, occasionally ate breakfast and never ate breakfast were 37.8, 41.6 and 20.6%, respectively. Finally, when measuring for sleep quality outcomes, 73.0% reported normal and good sleep quality.

**Table 2:** Comparison of demographic factors and prevalence of interpersonal sensitivity, anxiety symptoms and depression symptoms [*n* (%)].

Variables	Total (*n* = 12,922)	Interpersonal sensitivity				Anxiety symptoms				Depression symptoms			
		No	Yes	*χ* ^2^	*p*	No	Yes	*χ^2^*	*p*	No	Yes	*χ^2^*	*p*
**Gender**				9.231	0.002			23.314	<0.001			12.665	<0.001
Male	5,520 (42.7)	2,231 (40.4)	3,289 (59.6)			4,156 (75.3)	1,364 (24.7)			3,035 (55.0)	2,485 (45.0)		
Female	7,402 (57.3)	3,189 (43.1)	4,213 (56.9)			5,839 (78.9)	1,564 (21.1)			3,836 (51.8)	3,566 (48.2)		
**Age (years)**				68.124	<0.001			24.643	<0.001			84.311	<0.001
18–19	2,798 (21.7)	1,097 (39.2)	1701 (60.8)			2,190 (78.3)	608 (21.7)			1,497 (53.5)	1,301 (46.5)		
20–21	4,942 (38.2)	1935 (39.2)	3,007 (60.8)			3,710 (75.1)	1,232 (24.9)			2,402 (48.6)	2,540 (51.4)		
22–23	4,466 (34.6)	2025 (45.3)	2,441 (54.7)			3,523 (78.9)	943 (21.1)			2,530 (56.7)	1936 (43.3)		
≥24	716 (5.5)	363 (50.7)	353 (49.3)			572 (79.9)	144 (20.1)			442 (61.7)	274 (38.3)		
**Grade**				74.590	<0.001			53.727	<0.001			96.403	<0.001
Freshman	2,936 (22.7)	1,137 (38.7)	1799 (61.3)			2,338 (79.6)	598 (20.4)			1,611 (54.9)	1,325 (45.1)		
Sophomore	2,667 (20.6)	1,032 (38.7)	1,635 (61.3)			1946 (73.0)	721 (27.0)			1,246 (46.7)	1,421 (53.3)		
Junior	2,719 (21.0)	1,117 (41.1)	1,602 (58.9)			2,102 (77.3)	617 (22.7)			1,387 (51.0)	1,332 (49.0)		
Senior	2,937 (22.7)	1,306 (44.5)	1,631 (55.5)			2,256 (76.8)	681 (23.2)			1,615 (55.0)	1,322 (45.0)		
Five-grade	1,663 (12.9)	828 (49.8)	835 (50.2)			1,353 (81.4)	310 (18.6)			1,012 (60.9)	651 (39.1)		
**Learning status**				408.346	<0.001			309.273	<0.001			455.808	<0.001
Difficulty	559 (4.3)	116 (20.8)	443 (79.2)			301 (53.8)	258 (46.2)			161 (28.8)	398 (71.2)		
Normal	7,269 (56.3)	2,676 (36.8)	4,593 (63.2)			5,446 (74.9)	1823 (25.1)			3,477 (47.8)	3,792 (52.2)		
Good	4,231 (32.7)	2,108 (49.8)	2,123 (50.2)			3,511 (83.0)	720 (17.0)			2,622 (62.0)	1,609 (38.0)		
Excellent	863 (6.7)	520 (60.3)	343 (39.7)			737 (85.4)	126 (14.6)			611 (70.8)	252 (29.2)		
**Dormitory relationship**				584.943	<0.001			309.664	<0.001			344.758	<0.001
Poor	79 (0.6)	22 (27.8)	57 (72.2)			39 (49.4)	40 (50.6)			26 (32.9)	53 (67.1)		
Not very good	148 (1.1)	22 (14.9)	126 (85.1)			75 (50.7)	73 (49.3)			41 (27.7)	107 (72.3)		
Normal	1,625 (12.6)	401 (24.7)	1,224 (75.3)			1,048 (64.5)	577 (35.5)			623 (38.3)	1,002 (61.7)		
Good	6,515 (50.4)	2,489 (38.2)	4,026 (61.8)			5,094 (78.2)	1,421 (21.8)			3,357 (51.5)	3,158 (48.5)		
Excellent	4,555 (35.2)	2,486 (54.6)	2069 (45.4)			3,749 (82.1)	820 (17.9)			2,824 (62.0)	1731 (38.0)		
**Only child**				31.400	<0.001			4.294	0.038			29.495	<0.001
Yes	6,716 (52.0)	2,974 (44.3)	3,742 (55.7)			5,244 (78.1)	1,472 (21.9)			3,725 (55.5)	2,991 (44.5)		
No	6,206 (48.0)	2,446 (39.4)	3,760 (60.6)			4,751 (76.6)	1,455 (23.4)			3,146 (50.7)	3,060 (49.3)		
**Have breakfast**				134.169	<0.001			125.915	<0.001			364.550	<0.001
Never	2,668 (20.6)	961 (36.0)	1707 (64.0)			1886 (70.7)	782 (29.3)			1,091 (40.9)	1,577 (59.1)		
Sometimes	5,387 (41.6)	2,105 (39.2)	3,267 (60.8)			4,113 (76.6)	1,259 (23.4)			2,708 (50.4)	2,664 (49.6)		
Always	4,882 (37.8)	2,354 (48.2)	2,528 (51.8)			3,996 (81.9)	886 (18.1)			3,072 (62.9)	1810 (37.1)		
**Sleep quality**				1006.233	<0.001			1171.649	<0.001			1793.014	<0.001
Poor	468 (3.6)	204 (43.6)	264 (56.4)			270 (57.7)	198 (42.3)			186 (39.7)	282 (60.3)		
Not very good	1,099 (8.5)	238 (21.7)	861 (78.3)			560 (51.0)	539 (49.0)			215 (19.6)	884 (80.4)		
Normal	4,826 (37.3)	1,472 (30.5)	3,354 (69.5)			3,386 (70.2)	1,440 (29.8)			1892 (39.2)	2,934 (60.8)		
Good	4,608 (35.7)	2,224 (48.3)	2,384 (51.7)			4,002 (86.8)	606 (13.2)			3,022 (65.6)	1,586 (34.4)		
Excellent	1921 (14.9)	1,282 (66.7)	639 (33.3)			1777 (92.5)	144 (7.5)			1,556 (81.0)	365 (19.0)		

There was a significant discrepancy between male and female groups in severity of interpersonal sensitivity (*p* = 0.002), anxiety (*p* < 0.001), and depression symptoms (*p* < 0.001). Only child was less likely than non-only child for interpersonal sensitivity (*p* < 0.001), anxiety (*p* = 0.038) and depression symptoms (*p* < 0.001). All the other selected variables were still significant differences with interpersonal sensitivity, anxiety and depressive symptoms during COVID-19 lockdown (all *p* < 0.001).

### Association of influence factors with interpersonal sensitivity, anxiety and depressive symptoms

The associations of potential influence factors with interpersonal sensitivity, anxiety and depressive symptoms during COVID-19 lockdown were presented in Table 3.

**Table 3:** Logistic regression analysis of interpersonal sensitivity, anxiety symptoms and depression symptoms.

Variables	Interpersonal sensitivity			Anxiety symptoms			Depression symptoms		
	*B*	OR (95%CI)	*p*	*B*	OR (95%CI)	*p*	*B*	OR (95%CI)	*p*
**Gender**									
Male	0.15	1.16 (1.07–1.25)	<0.001	0.18	1.20 (1.10–1.31)	<0.001	−0.18	0.84 (0.77–0.90)	<0.001
Female	Reference								
**Age (years)**									
18–19	0.51	1.67 (1.33–2.10)	<0.001	0.34	1.41 (1.07–1.85)	0.014	0.51	1.67 (1.32–2.11)	<0.001
20–21	0.52	1.69 (1.38–2.06)	<0.001	0.34	1.40 (1.10–1.78)	0.006	0.55	1.73 (1.41–2.13)	<0.001
22–23	0.34	1.40 (1.17–1.67)	<0.001	0.17	1.19 (0.96–1.47)	0.116	0.31	1.37 (1.14–1.65)	<0.001
≥24	Reference								
**Grade**									
Freshman	0.30	1.35 (1.12–1.63)	0.002	−0.05	0.95 (0.76–1.19)	0.658	0.07	1.07 (0.89–1.30)	0.463
Sophomore	0.16	1.17 (0.99–1.39)	0.070	0.24	1.27 (1.04–1.56)	0.020	0.23	1.26 (1.06–1.50)	0.009
Junior	0.11	1.11 (0.95–1.30)	0.184	0.04	1.04 (0.86–1.26)	0.682	0.10	1.10 (0.94–1.29)	0.242
Senior	0.11	1.12 (0.98–1.28)	0.110	0.19	1.20 (1.02–1.42)	0.027	0.11	1.12 (0.97–1.29)	0.116
Five-grade	Reference								
**Learning status**									
Difficulty	1.29	3.62 (2.78–4.69)	<0.001	0.98	2.67 (2.04–3.49)	<0.001	1.18	3.24 (2.51–4.19)	<0.001
Normal	0.57	1.76 (1.51–2.06)	<0.001	0.32	1.37 (1.11–1.69)	0.003	0.52	1.69 (1.43–2.00)	<0.001
Good	0.24	1.27 (1.08–1.49)	0.003	0.07	1.07 (0.86–1.33)	0.535	0.20	1.22 (1.02–1.45)	0.028
Excellent	Reference								
**Dormitory relationship**									
Poor	0.87	2.38 (1.40–4.02)	<0.001	1.05	2.84 (1.75–4.63)	<0.001	0.82	2.27 (1.35–3.82)	0.002
Not very good	1.58	4.85 (3.04–7.75)	<0.001	1.03	2.81 (1.97–4.00)	<0.001	0.99	2.69 (1.82–3.98)	<0.001
Normal	1.05	2.86 (2.51–3.27)	<0.001	0.59	1.81 (1.58–2.08)	<0.001	0.59	1.80 (1.58–2.04)	<0.001
Good	0.56	1.75 (1.61–1.90)	<0.001	0.13	1.14 (1.03–1.26)	0.011	0.28	1.32 (1.21–1.44)	<0.001
Excellent	Reference								
**Only child**									
No	0.14	1.15 (1.07–1.24)	<0.001	0.05	1.05 (0.96–1.15)	0.267	0.10	1.11 (1.03–1.20)	0.009
Yes	Reference								
**Have breakfast**									
Never	0.21	1.23 (1.11–1.37)	<0.001	0.22	1.24 (1.10–1.40)	<0.001	0.56	1.74 (1.57–1.94)	<0.001
Sometimes	0.13	1.14 (1.05–1.25)	0.002	0.09	1.10 (0.99–1.22)	0.084	0.30	1.35 (1.23–1.47)	<0.001
Always	Reference								
**Sleep quality**									
Poor	0.67	1.96 (1.58–2.44)	<0.001	1.94	6.98 (5.40–9.03)	<0.001	1.61	5.01 (3.99–6.28)	<0.001
Not very good	1.66	5.26 (4.40–6.29)	<0.001	2.25	9.52 (7.70–11.77)	<0.001	2.59	13.37 (11.03–16.20)	<0.001
Normal	1.26	3.52 (3.12–3.96)	<0.001	1.48	4.41 (3.66–5.31)	<0.001	1.66	5.25 (4.60–6.00)	<0.001
Good	0.66	1.92 (1.72–2.16)	<0.001	0.59	1.81 (1.49–2.19)	<0.001	0.72	2.06 (1.80–2.35)	<0.001
Excellent	Reference								

OR, odds ratio; CI, confidence interval.

#### Factors associated with interpersonal sensitivity

In logistic regression models, male students had higher potential risk than females (OR = 1.16; 95% CI = 1.07–1.25). College students aged 18–19 years, 20–21 years and 22–23 years showed significant association with interpersonal sensitivity (OR = 1.67; 95% CI = 1.33–2.10; OR = 1.69; 95% CI = 1.38–2.06; OR = 1.40; 95% CI = 1.17–1.67). Freshmen were more likely to be associated with a higher risk of interpersonal sensitivity (OR = 1.35; 95% CI = 1.12–1.63). In particular, the risk of interpersonal sensitivity for college students who were in difficulty, normal or good in learning state was 3.62, 1.76, and 1.27 times (respectively) higher than those who were excellent. Dormitory relationship (poor, not very good, normal or good) was more likely to be associated with a higher risk of interpersonal sensitivity (OR = 2.38; 95% CI = 1.40–4.02; OR = 4.85; 95% CI = 3.04–7.75; OR = 2.86; 95% CI = 2.51–3.27; OR = 1.75; 95% CI = 1.61–1.90). Non-only child (OR = 1.15; 95% CI = 1.07–1.24) were highly associated with interpersonal sensitivity. Compared with those who always have breakfast, those who always skip or only have breakfast occasionally are with interpersonal sensitivity (OR = 1.23; 95% CI = 1.11–1.37; OR = 1.14; 95% CI = 1.05–1.25). Sleep quality (poor, not very good, normal or good) was more likely to be associated with a higher risk of interpersonal sensitivity (OR = 1.96; 95% CI = 1.58–2.44; OR = 5.26; 95% CI = 4.40–6.29; OR = 3.52; 95% CI = 3.12–3.96; OR = 1.92; 95% CI = 1.72–2.16).

#### Factors associated with anxiety symptoms

In the final model, male students had higher potential risk than females (OR = 1.20; 95% CI = 1.10–1.31). 18–19 years and 20–21 years were more likely to be associated with a higher risk of anxiety symptoms (OR = 1.41; 95% CI = 1.17–1.85; OR = 1.40; 95% CI = 1.10–1.78). Sophomores and seniors showed significant association with anxiety symptoms (OR = 1.27; 95% = 1.04–1.56; OR = 1.20; 95% CI = 1.02–1.42). Compared with college students in excellent learning state, those who reported difficulty and normal in learning had higher risk of anxiety (OR = 2.67; 95% CI = 2.04–3.49, OR = 1.37; 95% CI = 1.11–1.69). Dormitory relationship poor, not very good, normal or good was more likely to be associated with a higher risk of anxiety (OR = 2.84; 95% CI = 1.75–4.63; OR = 2.81; 95% CI = 1.97–4.00; OR = 1.81; 95% CI = 1.58–2.08; OR = 1.14; 95% CI = 1.03–1.26). The odds ratios of anxiety symptoms were 1.24 (95% CI = 1.10–1.40) for those who never eat breakfast when compared with those who always have breakfast. Sleep quality (poor, not very good, normal or good) was more likely to be associated with a higher risk of anxiety symptoms (OR = 6.98; 95% CI = 5.40–9.03; OR = 9.52; 95% CI = 7.70–11.77; OR = 4.41; 95% CI = 3.66–5.31; OR = 1.81; 95% CI = 1.49–2.19).

#### Factors associated with depression symptoms

Males were less likely to have depression symptoms compared to female (OR = 0.84; 95% CI = 0.77–0.90). College students aged 18–19 years, 20–21 years and 22–23 years showed significant association with depression symptoms (OR = 1.67; 95% CI = 1.32–2.11; OR = 1.73; 95% CI = 1.41–2.13; OR = 1.37; 95% CI = 1.14–1.65). Sophomores had worse mental health status than five-grade (OR = 1.26; 95% CI = 1.06–1.50). Compared with college students in excellent learning state, those who reported difficultly, normal and good in learning had higher risk of depression symptoms (OR = 3.24; 95% CI = 2.51–4.19; OR = 1.69; 95% CI = 1.43–2.00; OR = 1.22; 95% CI = 1.02–1.45). Dormitory relationship (poor, not very good, normal or good) was more likely to be associated with a higher risk of depression symptoms (OR = 2.27; 95% CI = 1.35–3.82; OR = 2.69; 95% CI = 1.82–3.98; OR = 1.80; 95% CI = 1.58–2.04; OR = 1.32; 95% CI = 1.21–1.44). The non-only child (OR = 1.11; 95% CI = 1.03–1.20) were highly associated with depression symptoms. Compared with students who always eat breakfast, those who always skip breakfast or only have breakfast occasionally are highly associated with depressive symptoms (OR = 1.74; 95% CI = 1.57–1.94; OR = 1.35; 95% CI = 1.23–1.47). Sleep quality (poor, not very good, normal or good) was more likely to be associated with a higher risk of depression symptoms (OR = 5.01; 95% CI = 3.99–6.28; OR = 13.37; 95% CI = 11.03–16.20; OR = 5.25; 95% CI = 4.60–6.00; OR = 2.06; 95% CI = 1.80–2.35).

## Discussion

This study is aimed to explore the prevalence of interpersonal sensitivity, anxiety, depression symptoms and related factors among Chinese college students during the COVID-19 campus lockdown. Our findings indicated that the prevalence rates of interpersonal sensitivity, anxiety and depression among Chinese college students are 58.1, 22.7, and 46.8%, respectively. A systematic review showed that the prevalence of depressive symptoms ranged from 14.6 to 48.3% and anxiety symptoms ranged from 6.33 to 50.9% (Xiong et al., 2020), and a meta-analysis also have reported the relevant range (22.6–36.3% for anxiety and 16.5–48.3% for depression) among the general population in China during the pandemic (Pappa et al., 2020). Overall, the prevalence of anxiety and depression in our findings are within the range of general people without lockdown. The mean of interpersonal sensitivity in this study was 2.11, higher than reports from Morocco during COVID-19 pandemic (Sfendla and Hadrya, 2020). Longitudinal analysis from Italy showed that phobic anxiety and depressive symptoms increased during lockdown as compared with a few weeks before the COVID-19 outbreak, whereas interpersonal sensitivity decreased, probably due to quarantine status (Castellini et al., 2021). However, the prevalence of anxiety of our study was lower than studies from Ethiopian (Simegn et al., 2021) and Ugandan (Najjuka et al., 2021). The prevalence of depression in the current study was lower than reports from Ugandan (Najjuka et al., 2021), while the current prevalence was higher than studies in Ethiopian (Simegn et al., 2021) among college students during the pandemic.

Lockdown had a significant effect at all ages (Commodari and La Rosa, 2020), with high school and college students having higher levels of negative emotions and sleep problems (Zhou et al., 2020a). Our findings indicated that the clinical probable positive cases for anxiety and depression were 4.2 and 13.1%, respectively. Anxiety and depression symptoms among high school students in China in response to the COVID-19 pandemic were 7.1 and 12.8%, respectively (Cao et al., 2022). Compared with the results of this study, anxiety symptoms were higher and depression symptoms were lower, which may be because high school students are facing a stricter college entrance examination stress in China. In addition, compared with high school students, college students are more likely to use the Internet for online communication and emotional expression (Wang and Zhao, 2020). However, compared with the isolation at home just 1 month after the outbreak of the epidemic (Tang et al., 2020), the prevalence of depression among college students has increased in our study. It may be that the early outbreak of the epidemic coincided with the Spring Festival holiday, college students stayed at home with their family support to benefit for their mental health.

According to our survey, females suffered more from depressive symptoms than males. This result was consistent with the previous studies which indicated that females had more depression disorders (Tesfaye Kelemu et al., 2020, Zhou et al., 2020b, Junaid Tahir et al., 2022). Previous study from Ethiopia showed that female students are prone to social role and economic support, so they are more likely to suffer from psychological disorders compared with their male counterparts (Tesfaye Kelemu et al., 2020; Woday Tadesse et al., 2021). Previous research has shown that there are more anxiety symptoms of among females (Zhou et al., 2020b). However, a previous study from mainland China indicated that male students have higher levels of anxiety than female students (Sun et al., 2020), which was consistent with our studies. The difference may be due to different coping styles, family expectations, social roles and Chinese culture (Liang et al., 2020, Sun et al., 2020). Noticeably, males were positively associated with interpersonal sensitivity during the COVID-19 lockdown. Different from our research conclusion, a study showed that there is no gender difference in interpersonal sensitivity of college students (Carney and Harrigan, 2003). The proportion of male students in our study is higher than female students, which may also exert a certain impact on the research results, so it needs to be cautious to explain. In addition, a study among Iranian medical students showed prevalence of depression has no significant difference in genders among the medical students (Nakhostin-Ansari et al., 2020). Therefore, it seems that gender was not the determining factor in the development of depression.

The results of the present study indicated that young students had more interpersonal sensitivity, anxiety and depression symptoms than students of other age. The findings are similar to those from a study conducted in Saudi Arabia (Albagmi et al., 2021) and in Spanish (Becerra-García et al., 2020). College students are still in their early adulthood, and their mental level is immature, which is prone to psychological obstacles and mental diseases (Shi et al., 2017; Auerbach et al., 2019). The stress brought about by the pandemic, together with the pressure of the course itself and the damage of interpersonal relationships, has become the risk of mental health problems.

The results of study show that compared with graduation students, the interpersonal sensitivity of the freshman students is more serious; sophomores and seniors suffer from more anxiety symptoms, and the depression symptoms of sophomore are relatively more serious. Similar to some research results, for freshmen, psychological problems usually occur due to interpersonal relationships (Fiorilli et al., 2019, Xu et al., 2022). The high interpersonal sensitivity of freshmen may also be related to the new environment in which they enter the university. In our study, we found that higher grades have lower risk of depression symptom than lower grades, which was inconsistent with previous study indicating that final year students had more severe depression (Tang et al., 2020). The potential reason may be is that compared with high-age college students, low-age college students are mentally immature, unfamiliar with interpersonal relationship and the implicit character of Chinese people. Sophomores who suffered the COVID-19 epidemic prior to enrollment, had a negative experience. The recurrence of the epidemic may once again cause their anxiety and depression symptoms. The recurrence of the epidemic may once again cause their anxiety and depression symptoms. In addition, the sophomore year is loaded with considerably more professional courses and frequent evaluations (Wang et al., 2021), which may be caused more anxiety symptoms. Senior students have higher anxiety symptoms, which may be caused by the pressure of internship, employment pressure and graduation.

In general, the participants who with better learning status had less symptoms of interpersonal sensitivity, anxiety and depression symptoms. Similar to previous research results, for college students, a study found that lower grades point average was more likely to experience anxiety and depressed (Nakhostin-Ansari et al., 2020). Poor academic performance and poor academic records during COVID-19 were highly associated with severe depression and anxiety and other mental problems (Hou et al., 2020, Peng et al., 2022). Uncertainty about the impact of the epidemic may increase their concerns about the prospect of their studies, such as the shift of offline courses to online courses. Due to the outbreak of COVID-19, many students are not suitable for non-traditional teaching, so they may be more prone to depression and anxiety symptoms (Chen et al., 2021).

During the period when large-scale gathering is not allowed, dormitories have replaced classrooms and become one of the important places for college students’ daily activities. Undoubtedly, the relationship between roommates has changed in this process. The results of this study show that the better the roommate relationship, the less interpersonal sensitivity, anxiety and depression symptoms. Depression related psychosocial characteristics are low social contact with roommates (Hokanson et al., 1989). Roommate communication is an important part of college students’ interpersonal communication. The quality of roommate relationship directly affects the quality of college students’ school life. In the lockdown environment of the pandemic, in a tense state, the relationship between roommates has become particularly close, interpersonal relationships have become more sensitive than ever, and psychological problems are more likely to occur.

The non-only child was highly associated with interpersonal sensitivity and depression symptoms. A study among Chinese high school students showed a similar result, non-only children are risk factors for depressive symptoms and PTSD, but in terms of anxiety symptoms, they are not significant (Cao et al., 2022). It may be that families with many children often cause psychological problems such as depression and relatively poor psychological stability due to the influence of brother sister relationship, economic relationship and other factors (Zhang and Yu, 2008). During the epidemic period, the family income may decline, and the economic support received by non-only children may decrease more than that of only children. In addition, due to lockdown, it is impossible to go home to visit relatives frequently, and the reduction in family ties may lead to the increase of the mental pressure of non-only children.

Among the college students living on campus lockdown, the low frequency of eating breakfast is a risk factor for interpersonal sensitivity, anxiety and depression. In pandemic, fear and anxiety caused individuals to skip breakfast and have less snacks, but more for lunch (Kaya et al., 2021). It may be that habits of eating breakfast have changed during campus lockdown because of student irregularity of schedules and difficulty to have breakfast on time.

Sleep quality was found to have a strong association with interpersonal sensitivity, anxiety and depression symptoms in the current study. On the whole, college students with poor sleep quality are more likely to suffer from emotional distress and psychological problems. These results were consistent with a previous study that found the lockdown had a deleterious effect on the sleep of pharmacy students in South India (Bhat et al., 2022), leading to a series of psychological problems. A study showed that Indian students delayed sleep time by 1 h during lockdown than pre-lockdown days (Prabhat et al., 2022). Besides the daily routine, the level of daily physical activity and the eating habits of students were affected by the lockdown management. And sudden shift of learning ways has potential impact on sleep hygiene as well (Bhat et al., 2022; Peng et al., 2022).

## Limitations

Several limitations were present in our study. Firstly, as the study was focused on college students, the results may not be applicable to other adults or the general population. Secondly, since the survey is a cross-sectional study clarify causality cannot be clarified. Thirdly, our study did not include relevant variables such as social media use (Vancini et al., 2022), nutrition, and daily physical exercise (Abdul Rahim et al., 2022). In the future, we should pay more attention to the life style during lockdown. Further longitudinal studies on the long-term impact of COVID-19 pandemic on college students are needed to extend this study.

## Conclusion

During the COVID-19 campus lockdown in China, the prevalence of psychological problems was at a high level among college students particularly for respondents who are younger and in lower grade, with poor dormitory relationship, poor sleep quality, and negative learning status and who often skip breakfast. These findings contribute to a better understanding of mental health problems during pandemics among college students, especially in terms of interpersonal sensitivity.

Under the situation of epidemic containment and normalization management, college students should pay attention to their mental health and cultivate their good habits, such as eating breakfast on time, taking more exercise, participating in non-gathering interpersonal activities, maintaining sufficient sleep time, maintaining good roommate relations, and actively requiring necessary psychological counseling.

## Data availability statement

The original contributions presented in the study are included in the article/supplementary material, further inquiries can be directed to the corresponding author.

## Ethics statement

The research was conducted according to the guidelines of the Declaration of Helsinki and approved by the Ethical Committee of Xuzhou Medical University. Written informed consent was obtained from all participants for their participation in this study.

## Author contributions

HX contributed to the conception and design of the study, the manuscript preparation, and the final revision. ZW performed the data analysis and drafted the initial manuscript. LP revised the manuscripts. PZ guided the statistical analysis process. YM investigated the data and established the databases. XL contributed to the conception and design of the study. All authors contributed to the article and approved the submitted version.

## Funding

This study was funded by the Industry-University Collaborative Education Project of the Higher Education Department of MOE (202101085008), and the Xuzhou Social Development Key R&D plan (KC21306).

## Conflict of interest

The authors declare that the research was conducted in the absence of any commercial or financial relationships that could be construed as a potential conflict of interest.

## Publisher’s note

All claims expressed in this article are solely those of the authors and do not necessarily represent those of their affiliated organizations, or those of the publisher, the editors and the reviewers. Any product that may be evaluated in this article, or claim that may be made by its manufacturer, is not guaranteed or endorsed by the publisher.
